# Effects of Anthropogenic Emissions from Different Sectors on PM_2.5_ Concentrations in Chinese Cities

**DOI:** 10.3390/ijerph182010869

**Published:** 2021-10-15

**Authors:** Jie Yang, Pengfei Liu, Hongquan Song, Changhong Miao, Feng Wang, Yu Xing, Wenjie Wang, Xinyu Liu, Mengxin Zhao

**Affiliations:** 1 Key Research Institute of Yellow River Civilization and Sustainable Development & Collaborative Innovation Center on Yellow River Civilization of Henan Province, Henan University, Kaifeng 475004, China; yangjie19881019@163.com (J.Y.); chhmiao@henu.edu.cn (C.M.); wjwang95@163.com (W.W.); 13917209663@163.com (X.L.); 2 Institute of Urban Big Data, College of Geography and Environmental Science, Henan University, Kaifeng 475004, China; f_wang108@126.com; 3 College of Geography and Environmental Science, Henan University, Kaifeng 475004, China; 4 Key Laboratory of Geospatial Technology for the Middle and Lower Yellow River Regions (Henan University), Ministry of Education, Kaifeng 475004, China; 5 Henan Key Laboratory of Integrated Air Pollution Control and Ecological Security, Henan University, Kaifeng 475004, China; 6 Henan Ecological and Environmental Monitoring Center, Zhengzhou 450046, China; nikkoyu@126.com; 7 Institute of Technology, Technology & Media University of Henan Kaifeng, Kaifeng 475004, China; x2733006863@163.com

**Keywords:** PM_2.5_ concentrations, anthropogenic emissions, emission sectors, GeoDetector model, China

## Abstract

PM_2.5_ pollution has gradually attracted people’s attention due to its important negative impact on public health in recent years. The influence of anthropogenic emission factors on PM_2.5_ concentrations is more complicated, but their relative individual impact on different emission sectors remains unclear. With the aid of the geographic detector model (GeoDetector), this study evaluated the impacts of anthropogenic emissions from different sectors on the PM_2.5_ concentrations of major cities in China. The results indicated that the influence of anthropogenic emissions factors with different emission sectors on PM_2.5_ concentrations exhibited significant changes at different spatial and temporal scales. Residential emissions were the dominant driver at the national annual scale, and the NO_X_ of residential emissions explained 20% (*q* = 0.2) of the PM_2.5_ concentrations. In addition, residential emissions played the leading role at the regional annual scale and during most of the seasons in northern China, and ammonia emissions from residents were the dominant factor. Traffic emissions play a leading role in the four seasons for MUYR and EC in southern China, MYR and NC in northern China, and on a national scale. Compared with primary particulate matter, secondary anthropogenic precursors have a more important effect on PM_2.5_ concentrations at the national or regional annual scale. The results can help to strengthen our understanding of PM_2.5_ pollution, improve PM_2.5_ forecasting models, and formulate more precise government control policy.

## 1. Introduction

Particulate matter (including PM_2.5_ and PM_10_, etc.), particularly fine particulate matter (PM_2.5_), is an air pollutant that exerts an important negative impact on public health [1,2,3,4]. Worldwide, more than 3.2 million people die prematurely due to outdoor particulate matter exposure each year [5]. In recent years, with the acceleration of China’s industrialization and urbanization and the continuous expansion of the urban scale, PM_2.5_ pollution has gradually attracted the attention of the government and academic circles [6,7,8].

Clarifying the mechanism of PM_2.5_ pollution is key to formulating effective pollution control policies. Natural geographic elements, including climate and topography, exert an important impact on PM_2.5_ [9,10,11,12,13,14,15,16]. Related studies have discussed meteorological factors, including temperature [17,18,19], humidity [20,21], wind speed [22,23], precipitation [24], atmospheric pressure [25], boundary layer height [26,27], solar radiation [28], and other factors that contribute to PM_2.5_. In addition to physical and geographic elements, human factors also exert a significant impact on PM_2.5_ concentrations [29]. Researchers have discussed the impact of many aspects on PM_2.5_ concentrations, such as land use [30,31], urbanization [32,33], transportation [34], economic development [35,36], and emissions premises [37,38,39,40,41]. Studies including [42] and others have studied the impact of reforestation and conversion of arable land on future air quality in the southern United States. The results showed that reforestation in the southeastern part of the United States led to a slight increase in summer PM_2.5_ in the southeastern United States, and the conversion of forest land to cultivated land led to an increase in annual PM_2.5_ concentrations. Studies including [43] and others have studied the impact of urban expansion on air quality in eastern China. The results have shown that intensive urbanization has an appropriate dilution effect on the concentrations of surface pollutants, but improper local emission control measures will aggravate regional haze pollution. Studies including [44] and others found that improper local emission control has been also found to have a great impact on light pollution among regional haze pollution. Studies including [45] and others have discussed the relationship between urbanization and other human factors, including clean energy consumption, emission reduction input, industry, and PM_2.5_ concentrations. The results have shown that the increase in PM_2.5_ concentrations is associated with urban areas. The protection of chemical and industrial growth is consistent, and increasing emissions reduction inputs and clean energy can slow the trend of increasing PM_2.5_ concentrations. Studies including [46] and others discussed the impact of social and economic development on major Chinese cities in terms of population density, economic growth, industrial base, industrial dust emissions, road density, trade openness, and energy consumption. The results showed that population density, industrial base, industrial dust emission, and road density have a significant positive effect on PM_2.5_ concentrations, while economic growth has a negative effect on PM_2.5_ concentrations. Studies including [35] and other studies have found that industrialization, urbanization, and economic growth in China have a positive correlation with PM_2.5_ concentrations. Studies including [47] and others found that land use/cover changes in PM_2.5_ concentrations showed a significant difference in different regions. Studies including [48] and others discussed the relationship between urban spatial structure and air quality in the United States, and found that proximate forests to urban areas reduce the number of AQI exceedance days. In addition, related researchers have integrated natural and human factors to discuss their influence on air quality [49,50]. Studies including [51] and others explored the impact of future climate change and anthropogenic emissions on the air quality in Portugal and Porto metropolitan areas in 2050 through different scenarios. The results showed that climate change and anthropogenic emissions have obvious temporal and spatial differences in the impact of air quality.

In summary, although existing research has made considerable progress in understanding the influence mechanism of PM_2.5_ concentrations, there remain some shortcomings. Existing studies mostly analyze the driving mechanism at a single temporal and spatial scale, and the differences of impact factors at different temporal and spatial scales are insufficiently considered. As far as anthropogenic emission factors are concerned, the impact mechanism of different sectors and different types of anthropogenic emissions on PM_2.5_ at different temporal and spatial scales is still unclear. This study focused on the entirety of China and analyzed the impact mechanism of emissions from different sectors on PM_2.5_ changes at different temporal and spatial scales. In addition, unlike most previous studies, which have only roughly characterized the humanistic driving mechanism through statistical data, this study focused more on the differences within the emission sector and explored the impact of anthropogenic emissions between different sectors on PM_2.5_ to clarify the difference in the impact of sector emissions on PM_2.5_ from a more detailed perspective.

Based on the emission inventory and site monitoring data, this study used the geographic detector method to explore the influence mechanism of the anthropogenic emission factors of different sectors on the PM_2.5_ concentrations. The research results will help researchers improve the accuracy of air quality forecasting models and also provide a basis and reference for government departments to formulate more accurate particulate matter emission government control policy.

## 2. Materials and Methods

### 2.1. Study Area

This study takes China as the research area and selects 366 major cities as samples to analyze the impact of the emissions of different sectors on the PM_2.5_ concentrations of major cities. Considering China’s natural and economic and social conditions and referring to the research of [52], the China region is divided into ten zones (Figure S1): the upper zone of the Yellow River (UYR); the middle and upper zone of the Pearl River (MUPR); the middle zone of the Yellow River (MYR); the northern coastal zone (NC); the northeast zone (NE); the middle and upper zone of the Yangtze River (MUYR); the southeast coastal zone (SC); the Xinjiang zone (XJ); the Qinghai-Tibetan Plateau (QTP); and the eastern coastal area (EC). Furthermore, part of the district is divided into two regions, the south and the north. NE, MYR, UYR, and NC belong to the northern region, while SC, MUPR, EC, and MUYR belong to the southern region.

### 2.2. Data

**Figure 1:**
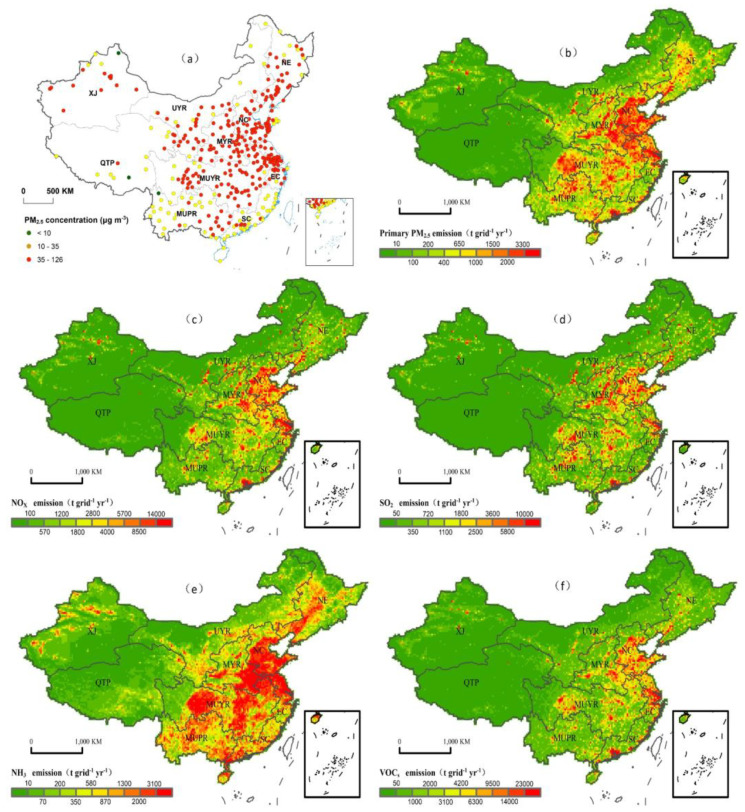
Maps of (**a**) annual average concentrations of PM_2.5_ (2015–2017) and (**b**) primary PM_2.5_ emissions, (**c**) NO_X_ emissions, (**d**) SO_2_ emissions, (**e**) NH_3_ emissions, and (**f**) VOC_S_ emissions from Chinese cities in 2016 with a resolution of 0.25 × 0.25.

The data used in this study include daily PM_2.5_ monitoring data from 366 cities (Figure S2) from 2015 to 2017 (National Environmental Monitoring Center). Based on this data, we can obtain the annual average concentrations (Figure 1a) from 2015 to 2017, as well as the seasonal average concentrations (Figures S2 and S3). The anthropogenic emission data of primary PM_2.5_, nitrogen oxides (NO_X_), volatile organic compounds (VOC_S_), sulfur dioxide (SO_2_) and ammonia (NH_3_) come from the 2016 Tsinghua University Emission Inventory (MEIC) (http://www.meicmodel.org/ (accessed on 10 December 2018)) (Figure 1b–f), the grid resolution is 0.25° × 0.25°, and the temporal resolution is month by month. The emission sector is divided into five sectors: industrial sources, agricultural sources, transportation sources, power sources and residential sources. The types of pollutants in each sector are as follows (Figures S4–S8): primary industry PM_2.5_, primary power PM_2.5_, primary residential PM_2.5_, primary transportation PM_2.5_, industrial NO_X_, power NO_X_, residential NO_X_, transportation NO_X_, industry VOC_S_, power VOC_S_, residential VOC_S_, transportation VOC_S_, agricultural NH_3_, industrial NH_3_, residential NH_3_, transportation NH_3_, industrial SO_2_, and power SO_2_.

### 2.3. GeoDetector Model

Geographic detectors are a set of statistical methods that use spatial similarity to detect the influence of independent variables on dependent variables and reveal the driving force behind the phenomenon. This method has no linear assumptions and has clear physical meaning. It includes 4 modules: the factor detection module, interaction function detection module, risk area detection module, and ecological detection module [53]. This study uses the factor detection module in GeoDetector to evaluate the contribution of pollutants emitted by different sectors to the PM_2.5_ concentrations of major cities in China. The factor detection module can express the degree of interpretation of the independent variable to the dependent variable through the *q* value. For specific methods, please refer to related research [54,55].

The factor detection module used in this article is used to detect the spatial differentiation of the dependent variable and quantify the degree of interpretation of a certain independent variable to the dependent variable, measured by the *q* value. Assume that the PM_2.5_ concentrations in the area in which the city is located are composed of daily emission data of 366 cities across the country. Assuming that there may be a factor that affects the concentrations of PM_2.5_, expressed as *A* = {*A_h_*}, *h* = 1, 2, …, *L*, *L* is the number of influencing factors, *A_h_* is the different types of influencing Factors A, and A-type h corresponds to one or more regional subsets of the city in space. To detect the spatial correlation between influencing Factor A and urban PM_2.5_ concentrations, the layers of influencing Factor A and the urban PM_2.5_ concentrations map are superimposed. In the h type of influencing Factor A, the discrete variance of urban PM_2.5_ concentrations is denoted as *σ_h_*^2^, and the degree of interpretation of PM_2.5_ concentrations by influencing Factor A can be expressed as:

(1) $$q=1-\frac{\sum_{h=1}^{L}N_{h}\sigma_{h}{}^{2}}{N\sigma^{2}}$$

In the formula, *q* is the explanatory power of urban PM_2.5_ concentrations influencing factors, and its value range is [0, 1]; *L* is the number of urban subregions divided by influencing factors, which this article uses equal interval classification to divide into 6 levels; h is a certain subarea; *N_h_* is the number of samples in a given subarea; *N* is the number of samples in the entire study area; *σ_h_*^2^ is the discrete variance of the PM_2.5_ concentrations in a given subregional city; *σ*^2^ is the variance of the PM_2.5_ concentrations in the entire regional city. If the *q* value of a certain influencing factor is higher, its explanatory power is stronger, and the influence of this factor on urban PM_2.5_ concentrations is stronger. Conversely, if the *q* value of a certain influencing factor is smaller, its explanatory power is weaker, and the influence of this factor on urban PM_2.5_ concentrations is weaker. In extreme cases, a *q* value of 0 indicates that the influencing factor and urban PM_2.5_ concentrations score have no relationship. A *q* value of 1 indicates that the influencing factors completely affect the distribution of urban PM_2.5_ concentrations.

## 3. Results

### 3.1. Analysis on the National Annual Scale

**Figure 2:**
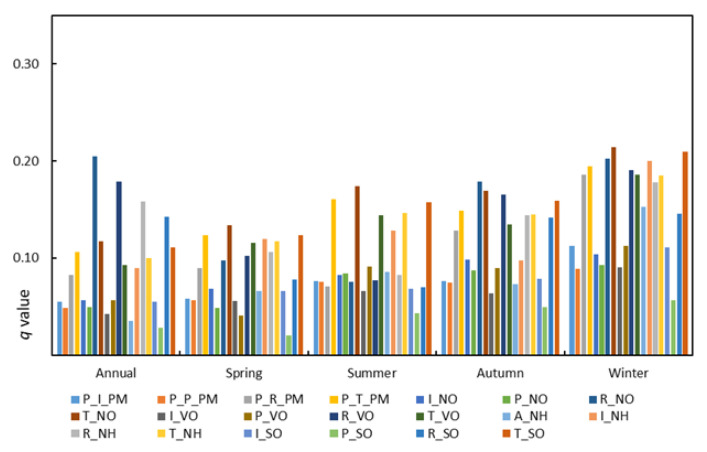
The *q* value of the driving factor on the national annual and seasonal scales. (P_I_PM denotes primary industry PM_2.5_; P_P_PM denotes primary power PM_2.5_; P_R_PM denotes primary residential PM_2.5_; P_T_PM denotes primary transportation PM_2.5_; I_NO denotes industrial NO_X_; P_NO denotes power NO_X_; R_NO denotes residential NO_X_; T_NO denotes transportation NO_X_; I_VO denotes industry VOC_S_; P_VO denotes power VOC_S_; R_VO denotes residential VOC_S_; T_VO denotes transportation VOC_S_; A_NH denotes agricultural NH_3_; I_NH denotes industrial NH_3_; R_NH denotes residential NH_3_; T_NH denotes transportation NH_3_; I_SO denotes industrial SO_2_; and P_SO denotes power SO_2_).

The driving factors of PM_2.5_ concentrations in all Chinese cities have significant annual changes on an annual scale. If the emission sectors are not considered, the primary two factors influencing the concentrations of PM_2.5_ on the national annual scale are NH and P_PM (Figure S9). If the emission sectors are considered, the dominant factors that affect PM_2.5_ concentrations on the national annual scale come from residential sources. Among them, the dominant factor is R_NO (*q* = 0.20), followed by R_VO (*q* = 0.18), R_NH (*q* = 0.16) and R_SO (*q* = 0.14) (Figure 2).

### 3.2. Analysis on the National Seasonal Scale

If the emission sectors are not taken into account, the dominant factors that affect the PM_2.5_ concentrations on the national scale in spring are NO and NH (Figure S9). In summer, these types of anthropogenic emissions have similar effects on PM_2.5_ concentrations on the national scale. Similar to summer, these types of anthropogenic emissions have similar effects on PM_2.5_ concentrations in autumn (except SO). However, the influence of NH and P_PM on PM_2.5_ concentrations are stronger than other factors in winter on the national scale.

If the emission sectors are considered, traffic sources play a leading role in the influence of PM_2.5_ concentrations compared with several other emission sources in spring on the national scale (Figure 2). The contribution of the influencing factors is ranked as follows: T_NO (*q* = 0.13), T_SO (*q* = 0.12), T_VO (*q* = 0.12). Similar to spring, the main driving factors come from traffic sources in summer. T_NO (*q* = 0.17) ranks first, followed by T_SO (*q* = 0.16) and T_NH (*q* = 0.15). In autumn, residential sources and traffic emissions play a leading role in PM_2.5_ concentrations and have similar effects on the national scale. R_NO (*q* = 0.18) and R_NO (*q* = 0.17) are the top two factors. Similar to autumn, residential sources and traffic sources in winter play a leading role in the PM_2.5_ concentrations and have similar effects, and the dominant factors are T_NO and T_SO.

### 3.3. Analysis on the Regional Annual Scale

If the emission sectors are not considered, these types of anthropogenic emissions have similar effects on PM_2.5_ concentrations in most regions on the annual scale (except MYR and NC) (Figure S10). NH is the dominant factor of PM_2.5_ concentrations in NC, while P_PM and NH3 are the two main factors on PM_2.5_ concentrations in MYR.

**Figure 3:**
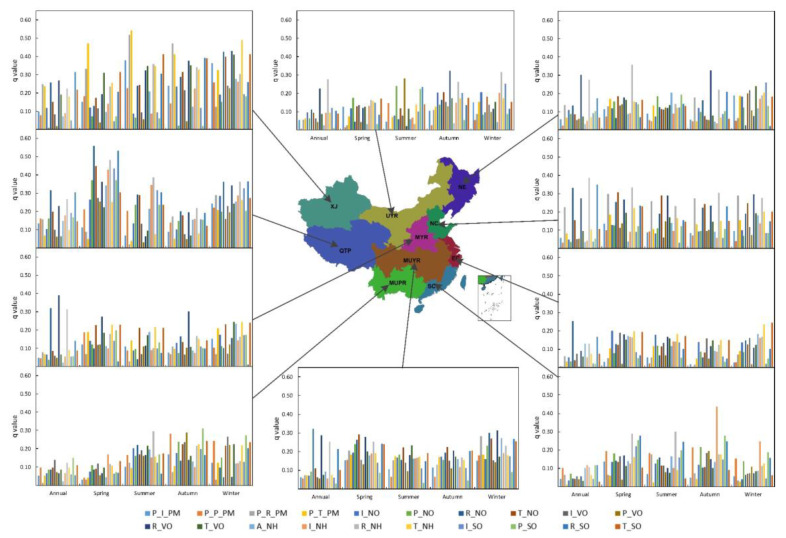
The *q* value of the driving factor on the regional annual and seasonal scales. (P_I_PM denotes primary industry PM_2.5_; P_P_PM denotes primary power PM_2.5_; P_R_PM denotes primary residential PM_2.5_; P_T_PM denotes primary transportation PM_2.5_; I_NO denotes industrial NO_X_; P_NO denotes power NO_X_; R_NO denotes residential NO_X_; T_NO denotes transportation NO_X_; I_VO denotes industry VOC_S_; P_VO denotes power VOC_S_; R_VO denotes residential VOC_S_; T_VO denotes transportation VOC_S_; A_NH denotes agricultural NH_3_; I_NH denotes industrial NH_3_; R_NH denotes residential NH_3_; T_NH denotes transportation NH_3_; I_SO denotes industrial SO_2_; and P_SO denotes power SO_2_).

If the emission sectors are taken into account, residential sources are the basic factors on PM_2.5_ concentrations in most Chinese urban areas (except MUPR) (Figure 3). The northern area is dominated by R_VO and R_NH, while some southern areas (EC and MUYR) are dominated by R_NO. For the XJ and QTP regions, the dominant factors affecting PM_2.5_ concentrations are R_SO and R_NO, respectively.

### 3.4. Analysis on the Regional Seasonal Scale

If the emission sectors are not considered, NH is the dominant factor in northern China and SC in spring, and NO is the dominant factor in southern EC and MUYR (Figure S10). In summer, NH is the dominant factor of PM_2.5_ concentrations in most northern regions (except UYR) and in southern MUYR, VO is the dominant factor in URY and SC, and SO is the dominant factor in MUPR. In autumn, P_PM is the dominant factor of PM_2.5_ concentrations in NE, MYR, and SC. NH is the dominant factor of PM_2.5_ concentrations in NC and MUYR, and the dominant factor of PM_2.5_ concentrations in MUPR and UYR is VO. In winter, however, NH is the dominant factor of PM_2.5_ concentrations in NC, MYR, XJ, EC, SC and MUYR. VO is the dominant factor of PM_2.5_ concentrations in MUPR and NE, and SO is the dominant factor of PM_2.5_ concentrations in UYR in winter.

If the emission sectors are considered, emissions from residential sources and traffic sources are the dominant factors affecting the change in PM_2.5_ concentrations in spring in most regions (Figure 3). NE, NC, MYR in the northern region and SC in the southern region are dominated by residential source emissions, while traffic emissions dominate in southern EC and MUYR. Similar to spring, summer residential and traffic sources remain the basic influencing factors for PM_2.5_ concentrations changes in most parts of the country. Among them, NC, MYR, and XJ in the north and EC and MUYR in the south are mainly due to traffic emissions, whereas residential source emissions play a leading role in the southern MUPR and SC. In autumn, residential source emissions are the dominant factor in the changes in PM_2.5_ concentrations in northern regions (UYR, NE, NC, MYR), with the R_VO factor being the primary influencing factor, followed by R_NH. In winter, traffic emissions are the dominant factor affecting the changes in PM_2.5_ concentrations in the XJ, MYR, and EC regions, whereas residential source emissions are the dominant factors in the UYR, NC, and MUYR regions.

## 4. Discussion

The concentrations of PM_2.5_ is affected by both natural and human factors, and anthropogenic emissions play an important role in its formation. Numerous studies have analyzed the formation of PM_2.5_ and the influencing factors of its concentrations [56,57], but few studies have focused on the impacts of these factors on PM_2.5_ concentrations from the perspective of sector emissions. In addition, the research results can provide a more accurate reference for formulating PM_2.5_ control policies. In this paper, five types of pollutants emitted by five sectors, including residential sources, power, transportation, industry, and agriculture, were selected as driving factors. Based on the GeoDetector model, the impact of emission factors on the PM_2.5_ concentrations at different temporal and spatial scales was analyzed. The study found obvious seasonal and regional changes in the impact of pollutant emissions from different sectors on PM_2.5_ concentrations in Chinese cities. These variations are due mainly to the temporal and spatial differences in the emission of different precursors and photochemical reactions. Of course, anthropogenic emissions are also the main factors influencing the temporal and spatial differences in PM_2.5_ concentrations in China and even at the global scale [58,59,60].

At the national scale, residential emissions are the main influencing factor of the PM_2.5_ concentrations changes through the whole year. This result also verifies the proposal by other researchers that residential emissions play an important and non-negligible role in air pollution control [61,62]. However, at the national seasonal scale, the main driving factors of PM_2.5_ concentrations show more significant changes. The impact of residential emissions on PM_2.5_ concentrations in autumn and winter is stronger than that in spring and summer. These differences may be due to the unfavorable meteorological conditions of the planetary boundary layer in autumn and winter, such as temperature inversion and relatively frequent weak surface wind speed; moreover, the heating of living spaces and biomass burning in autumn and winter may easily cause an increase in PM_2.5_ concentrations [10,11,63,64]. It is worth noting that ammonia emissions exert a stronger impact on PM_2.5_ in winter compared to the other three seasons regardless of whether sectoral emissions are considered. Since the emission of NH_3_ in winter is lower than in other seasons, its impact is also higher than in other seasons, indicating that NH_3_ plays a very important role in the production of PM_2.5_ in winter, which is consistent with previous studies [65,66]. Therefore, measures to limit NH_3_ emissions should be considered when reducing PM_2.5_ pollution in winter to achieve better pollution control effects.

At the regional scale, we found that the impact of residential emissions on PM_2.5_ concentrations is dominant in the northern region and part of the southern region (MUYR and EC) through the whole year. Moreover, residential emissions also play a leading role in most seasons in the northern region. This impact may be because coal combustion and biomass combustion accounted for a large proportion of residential sources in the northern region, and these sources exert a greater impact on PM_2.5_ concentrations, especially in spring, autumn and winter, which is consistent with existing studies [67,68,69]. In addition, the importance of residential source NH_3_ emissions has been neglected. Studies have shown that residential NH_3_ emissions exert a significant impact on PM_2.5_ concentrations at the regional annual scale and in most seasons in northern China. This finding is consistent with related studies. For example, NH_3_ emissions from urban residential sewage account for a relatively high proportion of total NH_3_ emissions and have an important impact on PM_2.5_ concentrations [39]. It is worth noting that traffic emissions play a leading role in the four seasons for MUYR and EC in southern China, MYR and NC in northern China, and on a national scale. China’s national statistics [70] show that from 1990 to 2012, the number of motor vehicles in China has increased 22 times in 22 years, and traffic emissions have become one of the largest sources of air pollution in China. On the other hand, this may be due to the fact that these areas are located in the central and eastern parts of China, with higher road network density, higher car ownership, and accelerated photochemical reactions under suitable weather conditions, which have an important impact on PM_2.5_ concentrations [7,71,72].

The effects of primary emissions and secondary precursors on PM_2.5_ concentrations have obvious differences at different temporal and spatial scales. At the annual scale, whether national or regional, primary PM_2.5_ emissions contribute less to the PM_2.5_ concentrations than secondary emissions. This finding is consistent with previous research, indicating that secondary emissions make an important contribution to PM_2.5_ concentrations [73]. At the national seasonal scale, the contribution of primary PM_2.5_ to the PM_2.5_ concentrations is equivalent to that of secondary emissions, and the dominant factor comes from the primary emission of PM_2.5_ from transport sources. However, on the regional seasonal scale, the contribution of primary PM_2.5_ to PM_2.5_ concentrations is significantly different from that of secondary precursor emissions. Therefore, when formulating PM_2.5_ control policies, it is necessary to consider the actual conditions of the region and use differentiated emission control policies in different regions and seasons.

This study comprehensively quantifies the impact of different sectors’ emissions on PM_2.5_ concentrations. The results of the study can help us understand the anthropogenic emission mechanism of PM_2.5_ pollution changes and provide a reference for follow-up research and the formulation of policies and measures for related sectors. However, there are some limitations. (1) Due to the uncertainties in the emission inventory adopted, our research results will also have corresponding uncertainties. Moreover, given data availability limitations, the current inventory data used is only for one year (2016); data from other years need to be analyzed in future work. (2) Given length restrictions and the previous research results that have been obtained, this article does not consider meteorological factors in its focus. In the future, it will be necessary to comprehensively consider the impact of land use/cover change, topography, and socioeconomic factors on PM_2.5_ concentrations. (3) Related research shows that the concentrations of PM_2.5_ and O_3_ sometimes fluctuate, which also makes it difficult to formulate pollution control policies. Future research will combine the two pollutants of PM_2.5_ and O_3_ for analysis, thereby clarifying the impact mechanisms and formulating a more accurate policy reference for the government to comprehensively control air pollution.

## 5. Conclusions

The results of this study show that the effects of emission factors among various sectors on PM_2.5_ concentrations present large regional and seasonal differences. Residential emissions were the dominant driver at the national annual scale, and also played the leading role at the regional annual scale and during most of the seasons in northern China. Residential NH_3_ emissions exert a significant impact on PM_2.5_ concentrations at the regional annual scale and in most seasons in northern China. Transportation emissions play a leading role in the four seasons for MUYR and EC in southern China, MYR and NC in northern China, and on a national scale. Compared with primary particulate matter, secondary anthropogenic precursors have a more important effect on PM_2.5_ concentrations at the national or regional annual scale.

The influence mechanism of pollutants discharged by different sectors on PM_2.5_ concentrations is more complicated, which also makes it difficult for us to clarify the contributions of various factors to PM_2.5_ concentrations. This study comprehensively and quantitatively assessed the impact of anthropogenic emissions from various sectors and their interactions on PM_2.5_ concentrations. Given the complex nonlinear relationship between PM_2.5_ concentrations and its influencing factors, this study will help us better understand the impact mechanism of PM_2.5_ pollution. Moreover, it will help researchers improve the accuracy of PM_2.5_ prediction models and help governments formulate more precise PM_2.5_ pollution control policies.

## Supplementary Materials

The following are available online at https://www.mdpi.com/article/10.3390/ijerph182010869/s1, Figure S1: Ten regions in China. Figure S2: Locations of air quality monitoring stations. Figure S3: Seasonal mean PM_2.5_ concentrations in spring (a), summer (b), autumn (c), and winter (d) in Chinese cities (2015–2017). Figure S4: Maps of (a) primary industry PM_2.5_ emissions, (b) primary power PM_2.5_ emissions, (c) primary resident PM_2.5_ emissions, and (d) primary transportation PM_2.5_ emissions from Chinese cities with different emission sectors in 2016 with resolution of 0.25° × 0.25°. Figure S5: Maps of (a) agriculture NH_3_ emissions, (b) power NH_3_ emissions, (c) resident NH_3_ emissions, and (d) transportation NH_3_ emissions from Chinese cities with different emission sectors in 2016 with resolution of 0.25° × 0.25°. Figure S6: Maps of (a) industry NH3 emissions, (b) power NH_3_ emissions, (c) resident NH_3_ emissions, and (d) transportation NH_3_ emissions from Chinese cities with different emission sectors in 2016 with resolution of 0.25° × 0.25°. Figure S7: Maps of (a) industry SO_2_ emissions, (b) power SO_2_ emissions, (c) resident SO_2_ emissions, and (d) transportation SO_2_ emissions from Chinese cities with different emission sectors in 2016 with resolution of 0.25° × 0.25°. Figure S8: Maps of (a) industry VOC_S_ emissions, (b) power VOC_S_ emissions, (c) resident VOC_S_ emissions, and (d) transportation VOC_S_ emissions from Chinese cities with different emission sectors in 2016 with resolution of 0.25° × 0.25°. Figure S9: Annual and seasonal *q* values of driving factors for PM_2.5_ concentrations between PM_2.5_ concentrations and impacting factors at the national scale (China). P_PM denotes primary PM_2.5_; NO denotes NO_X_; VO denotes VOCs; NH denotes NH_3_; SO denotes SO_2_. Figure S10. Annual and seasonal *q* values of driving factors for PM_2.5_ concentrations between PM_2.5_ concentrations and impacting factors at the regional scale (China). P_PM denotes primary PM_2.5_; NO denotes NO_X_; VOCs VO denotes VOCs; NH denotes NH_3_; SO denotes SO_2_. Table S1: Effect of various anthropogenic factors with different emission sectors on PM_2.5_ concentrations in China in 2016. Table S2: Effect of various anthropogenic factors with different emission sectors on PM_2.5_ concentrations throughout the whole year at the regional scale. Table S3: Effect of various anthropogenic factors with different emission sectors on PM_2.5_ concentrations in spring at the regional scale. Table S4: Effect of various anthropogenic factors with different emission sectors on PM_2.5_ concentrations in summer at the regional scale. Table S5: Effect of various anthropogenic factors with different emission sectors on PM_2.5_ concentrations in autumn at the regional scale. Table S6: Effect of various anthropogenic factors with different emission sectors on PM_2.5_ concentrations in winter at the regional scale.

## Abbreviations

PM_2.5_	fine particulate matter
NOx	nitrogen oxides
VOCs	volatile organic compounds
SO_2_	sulfur dioxide
NH_3_	ammonia
UYR	the upper zone of the Yellow River
MUPR	the middle and upper zone of the Pearl River
MYR	the middle zone of the Yellow River
NC	the northern coastal zone
NE	the northeast zone
MUYR	the middle and upper zone of the Yangtze River
SC	the southeast coastal zone
XJ	the Xinjiang zone
QTP	the Qinghai-Tibetan Plateau
EC	the eastern coastal area
