# Supplementary material for: Effects of Anthropogenic Emissions from Different Sectors on PM2.5 Concentrations in Chinese Cities
Source: Int J Environ Res Public Health. 2021 Oct 15;18(20):10869. doi: 10.3390/ijerph182010869 (PMC8535752; doi:10.3390/ijerph182010869)
Supplement: Supplementary file 1 [file ijerph-18-10869-s001.zip › ijerph-1399606-supplementary.pdf]

# Supplementary Material

# Effects of anthropogenic emissions from different sectors on PM2.5 concentrations in Chinese cities

**Jie Yang [1], Pengfei Liu [1,2,5,*†], Hongquan Song [2,3,4**], Changhong Miao [1,5], Feng wang [2,3,4], Yu Xing [6], Wenjie Wang [1], Xinyu Liu [1], and Mengxin Zhao [7]**

1 Key Research Institute of Yellow River Civilization and Sustainable Development & Collaborative Innovation Center on Yellow River Civilization of Henan Province, Henan University, Kaifeng, Henan 475004, China;
2 Institute of Urban Big Data, College of Geography and Environmental Science, Henan University, Kaifeng, Henan 475004, China;
3 Key Laboratory of Geospatial Technology for the Middle and Lower Yellow River Regions (Henan University), Ministry of Education, Kaifeng, Henan 475004, China
4 Henan Key Laboratory of Integrated Air Pollution Control and Ecological Security, Henan University, Kaifeng, Henan 475004, China
5 College of Geography and Environmental Science, Henan University, Kaifeng, Henan 475004, China;
6 Henan Ecological and Environmental Monitoring Center, Zhengzhou, Henan 450000, China;
7 Institute of Technology, Technology & media university of Henan Kaifeng, Kaifeng, Henan475004, China;
* Correspondence:
† These authors contributed equally to this work and should be considered co-first authors.

## List of Figures

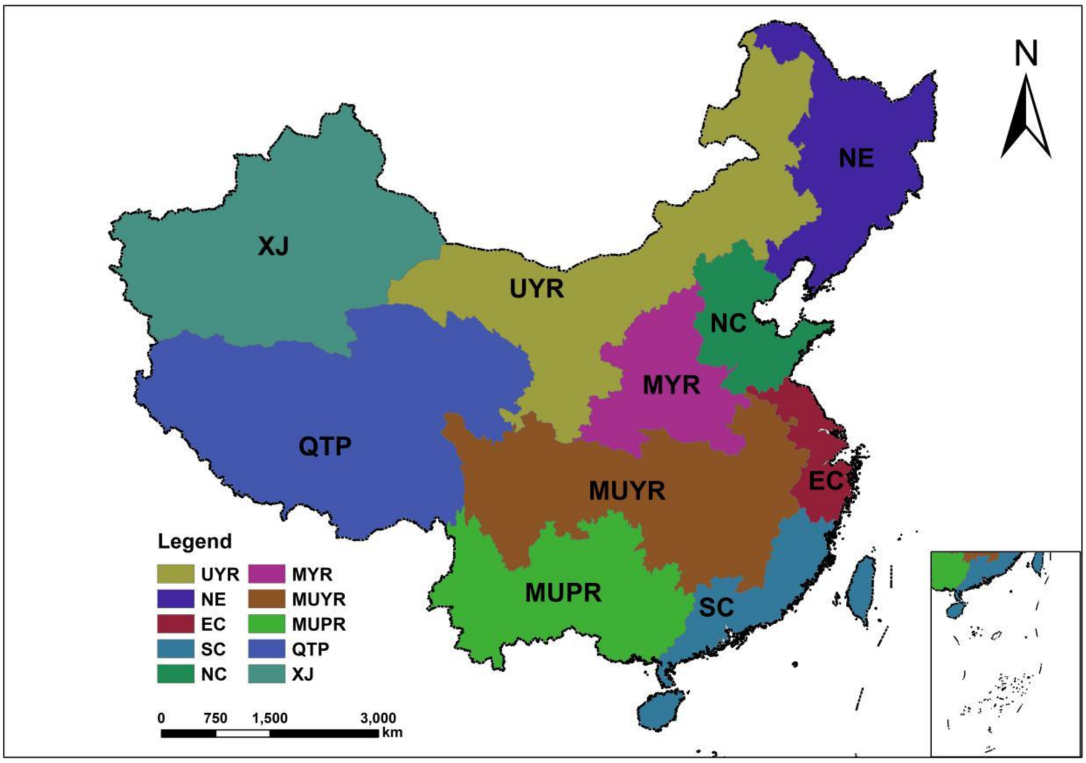


**Figure S1.** Ten regions in China, including Northeastern Area (NE), Northern Coastal Area (NC), Eastern Coastal Area (EC), Southeastern Coastal Area (SC), the Middle Reaches of the Yellow River Area (MYR), the Middle and Upper Reaches of the Yangtze River Area (MUYR), the Middle and Upper Reaches of the Pearl River Area (MUPR), the Upper Reaches of the Yellow

River Area (UYR), Xinjiang Area (XJ), and Qinghai-Tibetan Plateau Area (QTP) (Li et al., 2019).

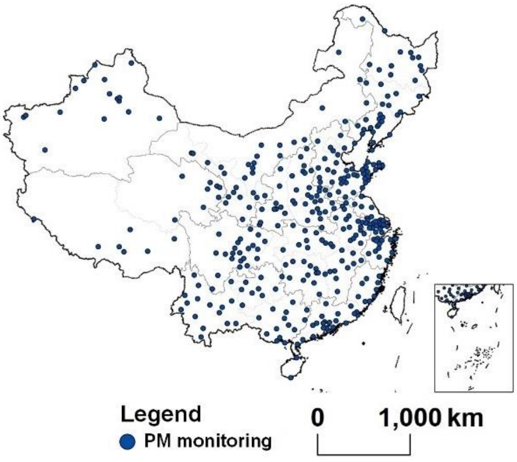


**Figure S2.** Locations of air quality monitoring stations (Li et al., 2019).

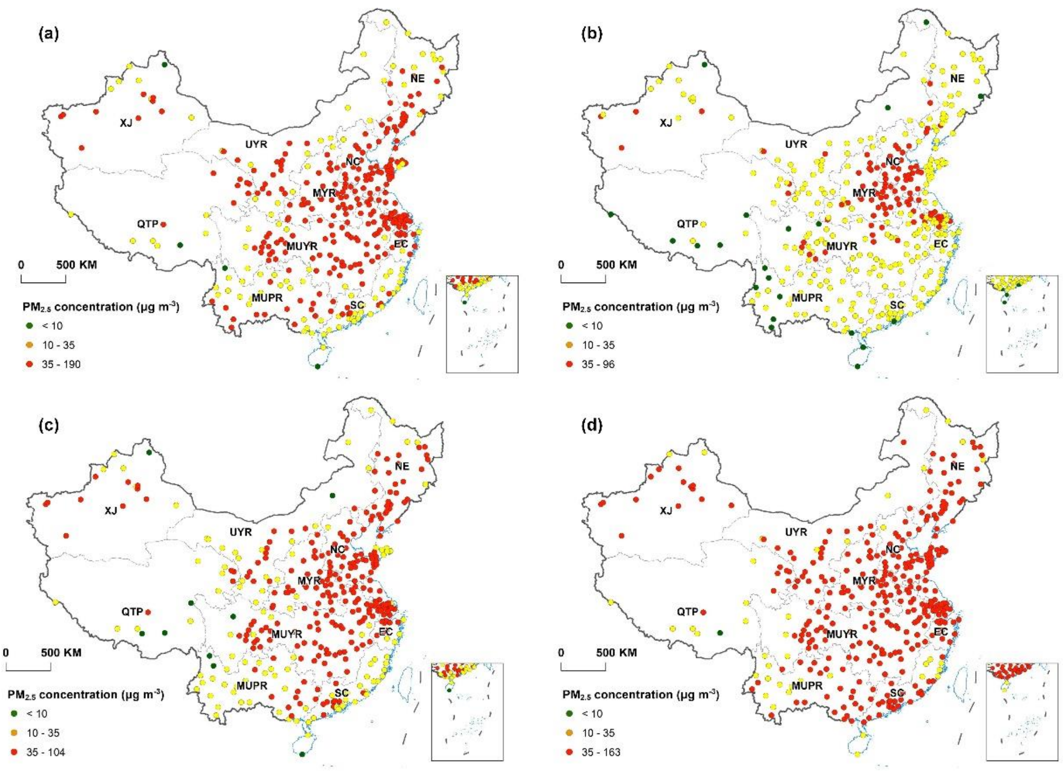


**Figure S3.** Seasonal mean PM2.5 concentrations in spring (a), summer (b), autumn (c), and winter (d) in Chinese cities (2015-2017).

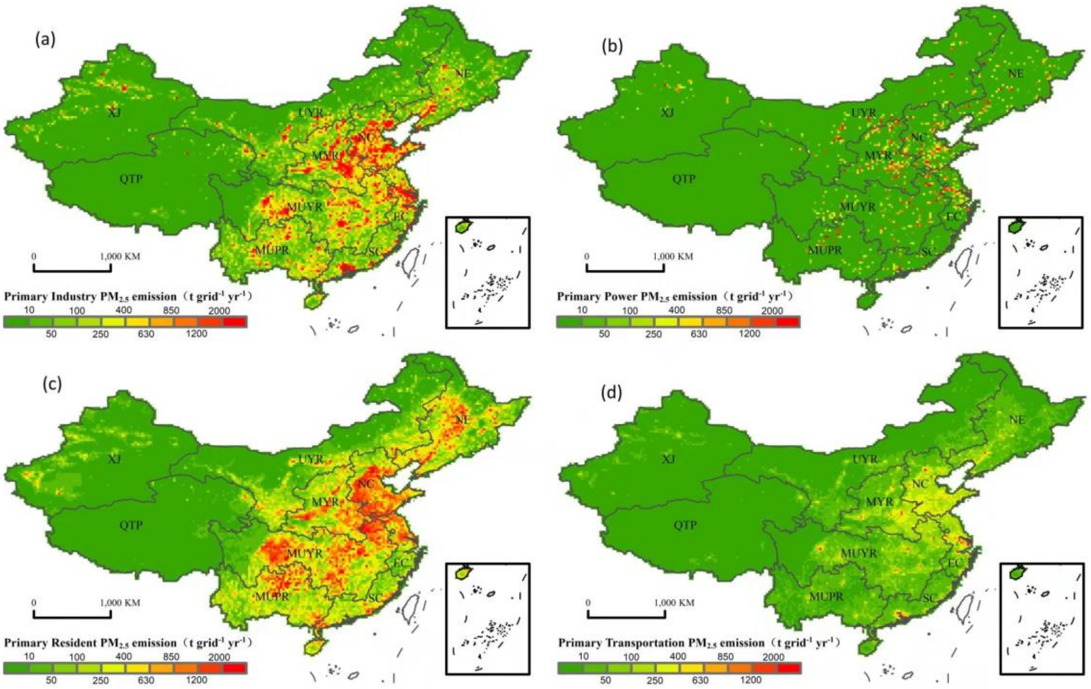


**Figure S4.** Maps of (a) Primary Industry $PM_{2.5}$ emissions, (b) Primary Power $PM_{2.5}$ emissions, (c) Primary Resident $PM_{2.5}$ emissions, and (d) Primary Transportation $PM_{2.5}$ emissions from Chinese cities with different emission sectors in 2016 with resolution of 0.25° × 0.25°.

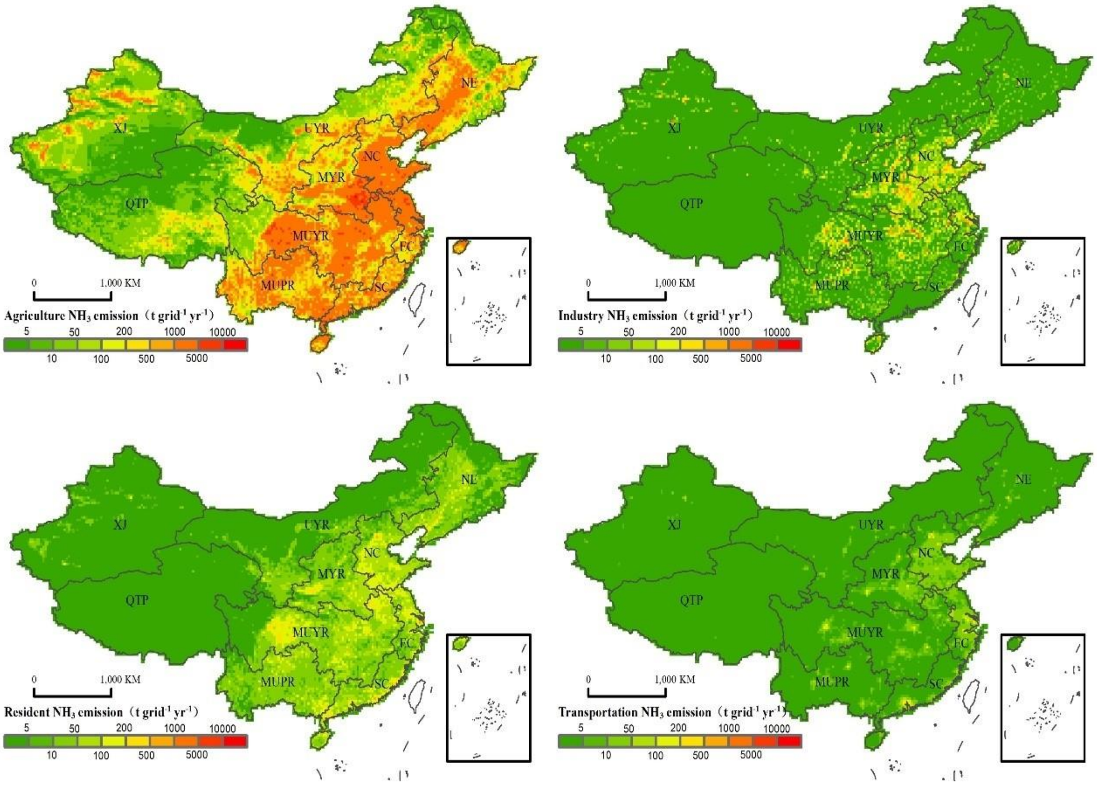

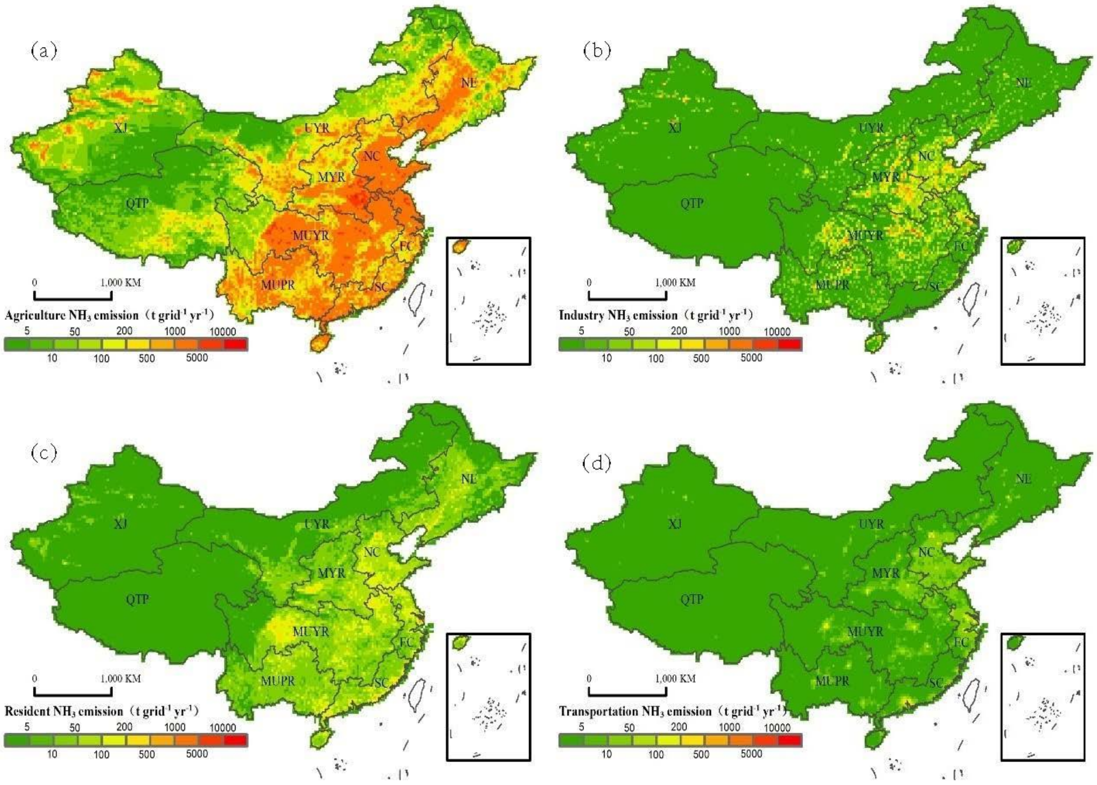


**Figure S5.** Maps of (a) Agriculture $NH_3$ emissions (b) Power $NH_3$ emissions, (c) Resident $NH_3$ emissions, and (d) Transportation $NH_3$ emissions from Chinese cities with different emission sectors in 2016 with resolution of 0.25° × 0.25°.

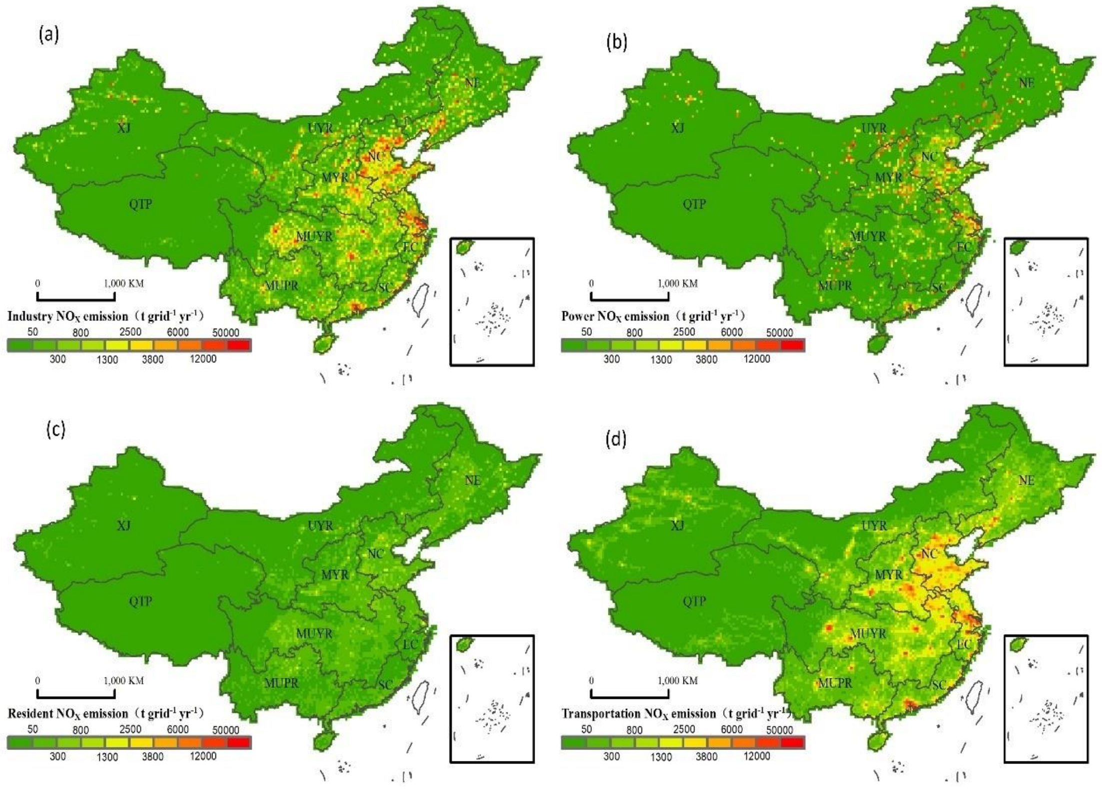


**Figure S6.** Maps of (a) Industry $NH_3$ emissions (b) Power $NH_3$ emissions, (c) Resident $NH_3$ emissions, and (d) Transportation $NH_3$ emissions from Chinese cities with different emission sectors in 2016 with resolution of 0.25° × 0.25°.

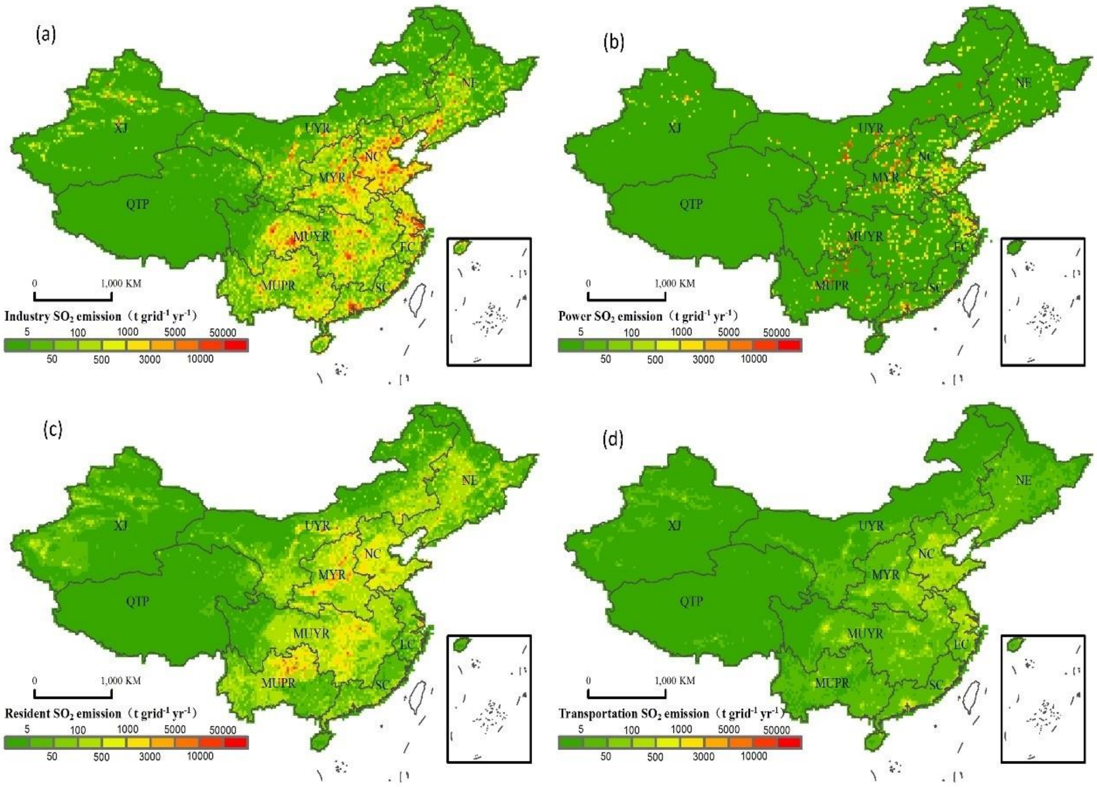


**Figure S7.** Maps of (a) Industry $SO_2$ emissions (b) Power $SO_2$ emissions, (c) Resident $SO_2$ emissions, and (d) Transportation $SO_2$ emissions from Chinese cities with different emission sectors in 2016 with resolution of 0.25° × 0.25°.

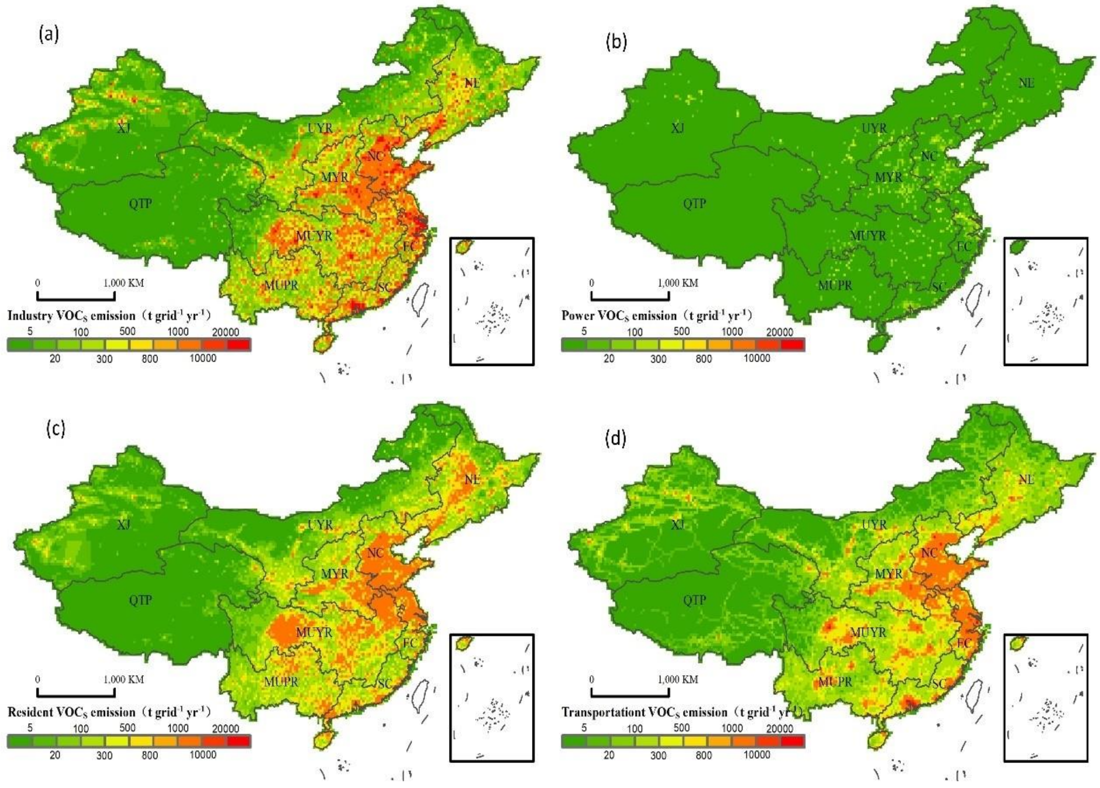


**Figure S8.** Maps of (a) Industry $VOC_S$ emissions (b) Power $VOC_S$ emissions, (c) Resident $VOC_S$ emissions, and (d) Transportation $VOC_S$ emissions from Chinese cities with different emission sectors in 2016 with resolution of 0.25° × 0.25°.

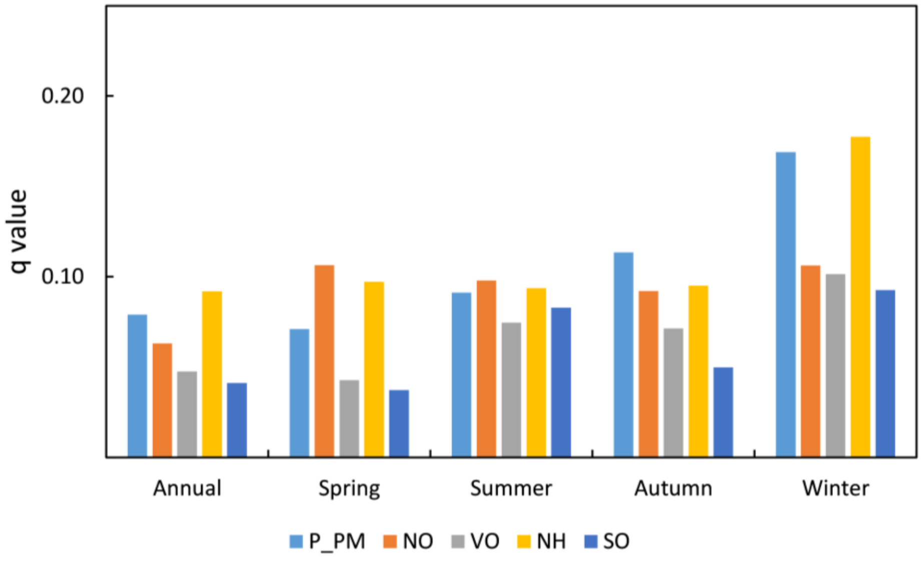


**Figure S9.** Annual and seasonal *q* values of driving factors for $PM_{2.5}$ concentrations between $PM_{2.5}$ concentrations and impacting factors at the national scale (China). P_PM denotes Primary $PM_{2.5}$; NO denotes $NO_X$; VOCs VO denotes VOCs; NH denotes $NH_3$; SO denotes $SO_2$

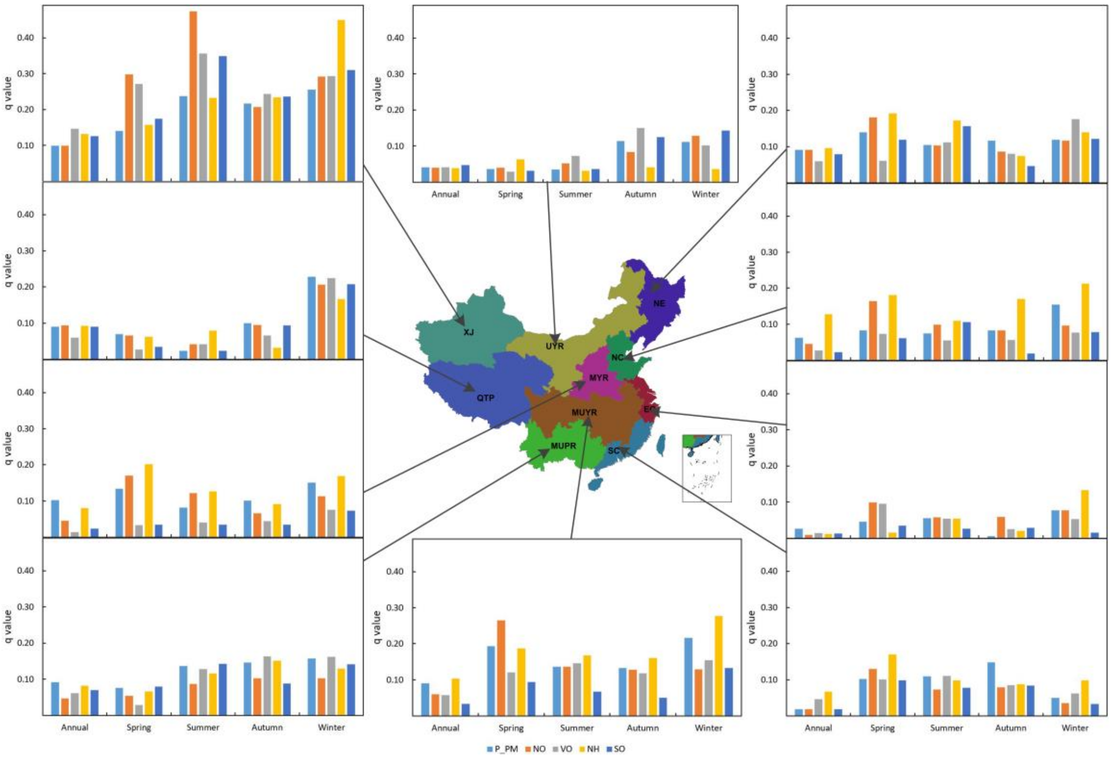


**Figure S10.** Annual and seasonal *q* values of driving factors for $PM_{2.5}$ concentrations between $PM_{2.5}$ concentrations and impacting factors at the regional scale (China). P_PM denotes Primary $PM_{2.5}$; NO denotes $NO_X$; VOCs VO denotes VOCs; NH denotes $NH_3$; SO denotes $SO_2$

**Table S1.** Effect of various anthropogenic factors with different emission sectors on $PM_{2.5}$ concentrations in China in 2016, value with *** is significant at the 0.01 level; value with ** is significant at the 0.05 level; value with * is significant at the 0.1 level.

| Time | Year | Spring | Summer | Autumn | Winter |
|---|---|---|---|---|---|
| P_I_PM | 0.05*** | 0.06*** | 0.08*** | 0.08*** | 0.11*** |
| P_P_PM | 0.05*** | 0.06*** | 0.08*** | 0.07*** | 0.09*** |
| P_R_PM | 0.08*** | 0.09*** | 0.07*** | 0.13*** | 0.19*** |
| P_T_PM | 0.11*** | 0.12*** | 0.16*** | 0.15*** | 0.19*** |
| I_NO | 0.06*** | 0.07*** | 0.08*** | 0.10*** | 0.10*** |
| P_NO | 0.05*** | 0.05*** | 0.08*** | 0.09*** | 0.09*** |
| R_NO | 0.20*** | 0.10*** | 0.08*** | 0.18*** | 0.20*** |
| T_NO | 0.12*** | 0.13*** | 0.17*** | 0.17*** | 0.21*** |
| I_VO | 0.04*** | 0.06*** | 0.07*** | 0.06*** | 0.09*** |
| P_VO | 0.06*** | 0.04*** | 0.09*** | 0.09*** | 0.11*** |
| R_VO | 0.18*** | 0.10*** | 0.08*** | 0.17*** | 0.19*** |
| T_VO | 0.09*** | 0.12*** | 0.14*** | 0.13*** | 0.19*** |
| A_NH | 0.04*** | 0.07*** | 0.09*** | 0.07*** | 0.15*** |
| I_NH | 0.09*** | 0.12*** | 0.13*** | 0.10*** | 0.20*** |
| R_NH | 0.16*** | 0.11*** | 0.08*** | 0.14*** | 0.18*** |
| T_NH | 0.10*** | 0.12*** | 0.15*** | 0.14*** | 0.19*** |
| I_SO | 0.05*** | 0.07*** | 0.07*** | 0.08*** | 0.11*** |
| P_SO | 0.03*** | 0.02*** | 0.04*** | 0.05*** | 0.06*** |
| R_SO | 0.14*** | 0.08*** | 0.07*** | 0.14*** | 0.15*** |
| T_SO | 0.11*** | 0.12*** | 0.16*** | 0.16*** | 0.21*** |

**Table S2.** Effect of various anthropogenic factors with different emission sectors on $PM_{2.5}$ concentration throughout the whole year at the regional scale, value with *** is significant at the 0.01 level; value with ** is significant at the 0.05 level; value with * is significant at the 0.1 level.

| Region | EC | MUPR | MUYR | MYR | NC | NE | QTP | SC | UYR | XJ |
|---|---|---|---|---|---|---|---|---|---|---|
| Year | 2016 | 2016 | 2016 | 2016 | 2016 | 2016 | 2016 | 2016 | 2016 | 2016 |
| P_I_PM | 0.01 | 0.05*** | 0.06*** | 0.05*** | 0.06*** | 0.06*** | 0.13*** | 0.04*** | 0.06*** | 0.10*** |
| P_P_PM | 0.00 | 0.10*** | 0.06*** | 0.05*** | 0.03*** | 0.03*** | 0.16*** | 0.10*** | 0.01 | 0.08*** |
| P_R_PM | 0.06*** | 0.01*** | 0.07*** | 0.08*** | 0.23*** | 0.14*** | 0.16*** | 0.06*** | 0.06*** | 0.25*** |
| P_T_PM | 0.03*** | 0.05*** | 0.07*** | 0.07*** | 0.08*** | 0.07*** | 0.07*** | 0.01 | 0.06*** | 0.24*** |
| I_NO | 0.06*** | 0.07*** | 0.07*** | 0.07*** | 0.05*** | 0.11*** | 0.10*** | 0.03*** | 0.10*** | 0.12*** |
| P_NO | 0.03*** | 0.09*** | 0.09*** | 0.04*** | 0.04*** | 0.09*** | 0.16*** | 0.07*** | 0.06*** | 0.01 |
| R_NO | 0.25*** | 0.09*** | 0.32*** | 0.32*** | 0.33*** | 0.14*** | 0.31*** | 0.05*** | 0.11*** | 0.26*** |
| T_NO | 0.04*** | 0.10*** | 0.11*** | 0.09*** | 0.16*** | 0.09*** | 0.20*** | 0.04*** | 0.09*** | 0.15*** |
| I_VO | 0.07*** | 0.14*** | 0.06*** | 0.06*** | 0.05*** | 0.05*** | 0.10*** | 0.06*** | 0.06*** | 0.08*** |
| P_VO | 0.00 | 0.07*** | 0.06*** | 0.05*** | 0.05*** | 0.06*** | 0.06*** | 0.04*** | 0.04*** | 0.02* |
| R_VO | 0.09*** | 0.07*** | 0.29*** | 0.39*** | 0.27*** | 0.30*** | 0.23*** | 0.06*** | 0.23*** | 0.27*** |
| T_VO | 0.06*** | 0.09*** | 0.08*** | 0.07*** | 0.10*** | 0.07*** | 0.07*** | 0.03*** | 0.09*** | 0.19*** |
| A_NH | 0.13*** | 0.02*** | 0.09*** | 0.02*** | 0.03*** | 0.03*** | 0.15*** | 0.10*** | 0.03** | 0.07*** |
| I_NH | 0.06*** | 0.07*** | 0.06*** | 0.06*** | 0.04*** | 0.05*** | 0.18*** | 0.12*** | 0.09*** | 0.09*** |
| R_NH | 0.13*** | 0.12*** | 0.25*** | 0.31*** | 0.39*** | 0.28*** | 0.27*** | 0.11*** | 0.28*** | 0.22*** |
| T_NH | 0.08*** | 0.10*** | 0.07*** | 0.09*** | 0.11*** | 0.07*** | 0.10*** | 0.06*** | 0.09*** | 0.18*** |
| I_SO | 0.02*** | 0.06*** | 0.06*** | 0.06*** | 0.04*** | 0.09*** | 0.19*** | 0.04*** | 0.12*** | 0.05*** |
| P_SO | 0.02** | 0.15*** | 0.03*** | 0.06*** | 0.05*** | 0.10*** | 0.17*** | 0.12*** | 0.01 | 0.01 |
| R_SO | 0.17*** | 0.06*** | 0.22*** | 0.14*** | 0.35*** | 0.17*** | 0.30*** | 0.12*** | 0.11*** | 0.32*** |
| T_SO | 0.08*** | 0.11*** | 0.10*** | 0.09*** | 0.11*** | 0.07*** | 0.15*** | 0.03*** | 0.09*** | 0.22*** |

**Table S3.** Effect of various anthropogenic factors with different emission sectors on $PM_{2.5}$ concentrations in spring at the regional scale, value with *** is significant at the 0.01 level; value with ** is significant at the 0.05 level; value with * is significant at the 0.1 level.

| Region | EC | MUPR | MUYR | MYR | NC | NE | QTP | SC | UYR | XJ |
|---|---|---|---|---|---|---|---|---|---|---|
| Season | spring | spring | spring | spring | spring | spring | spring | spring | spring | spring |
| P_I_PM | 0.03 | 0.03 | 0.15*** | 0.12*** | 0.13*** | 0.12*** | 0.11 | 0.14*** | 0.13*** | 0.15*** |
| P_P_PM | 0.00 | 0.04 | 0.16*** | 0.19*** | 0.10*** | 0.07*** | 0.21*** | 0.19*** | 0.02 | 0.18*** |
| P_R_PM | 0.05 | 0.03 | 0.21*** | 0.07*** | 0.30*** | 0.13*** | 0.09 | 0.07 | 0.03 | 0.33*** |
| P_T_PM | 0.10*** | 0.04 | 0.18*** | 0.19*** | 0.18*** | 0.17*** | 0.05 | 0.06 | 0.07 | 0.47*** |
| I_NO | 0.20*** | 0.07 | 0.19*** | 0.14*** | 0.13*** | 0.12*** | 0.27*** | 0.13*** | 0.12** | 0.12*** |
| P_NO | 0.08 | 0.11 | 0.24*** | 0.12*** | 0.13*** | 0.16*** | 0.37*** | 0.18*** | 0.18*** | 0.07* |
| R_NO | 0.13 | 0.09* | 0.26*** | 0.10*** | 0.25*** | 0.07 | 0.56*** | 0.14*** | 0.05 | 0.13*** |
| T_NO | 0.12*** | 0.09* | 0.29*** | 0.23*** | 0.31*** | 0.18*** | 0.45*** | 0.13*** | 0.13** | 0.17*** |
| I_VO | 0.19*** | 0.12** | 0.16*** | 0.12*** | 0.11*** | 0.14*** | 0.28*** | 0.17*** | 0.14** | 0.12*** |
| P_VO | 0.02 | 0.06 | 0.13*** | 0.12*** | 0.14*** | 0.14*** | 0.26*** | 0.04 | 0.04 | 0.04 |
| R_VO | 0.18*** | 0.07 | 0.28*** | 0.27*** | 0.27*** | 0.18*** | 0.36*** | 0.17*** | 0.12 | 0.19*** |
| T_VO | 0.15*** | 0.10** | 0.20*** | 0.19*** | 0.20*** | 0.17*** | 0.22*** | 0.11* | 0.13** | 0.31*** |
| A_NH | 0.17*** | 0.05 | 0.18*** | 0.11*** | 0.04 | 0.09 | 0.34*** | 0.14*** | 0.03 | 0.10** |
| I_NH | 0.17*** | 0.17*** | 0.19*** | 0.10*** | 0.09** | 0.09 | 0.43*** | 0.12 | 0.13 | 0.14*** |
| R_NH | 0.17*** | 0.12*** | 0.25*** | 0.18*** | 0.33*** | 0.36*** | 0.48*** | 0.29*** | 0.17*** | 0.24*** |
| T_NH | 0.20*** | 0.11* | 0.19*** | 0.23*** | 0.22*** | 0.16*** | 0.25*** | 0.16*** | 0.16*** | 0.26*** |
| I_SO | 0.09* | 0.06 | 0.14*** | 0.14*** | 0.09** | 0.15*** | 0.43*** | 0.22 | 0.15*** | 0.05 |
| P_SO | 0.05 | 0.07 | 0.09*** | 0.20*** | 0.12*** | 0.14*** | 0.37*** | 0.25 | 0.03 | 0.07* |
| R_SO | 0.07 | 0.07 | 0.24*** | 0.03 | 0.24*** | 0.07* | 0.53*** | 0.28 | 0.08* | 0.21*** |
| T_SO | 0.20*** | 0.13*** | 0.24*** | 0.23*** | 0.23*** | 0.17*** | 0.31*** | 0.10** | 0.17*** | 0.32*** |

**Table S4.** Effect of various anthropogenic factors with different emission sectors on $PM_{2.5}$ concentrations in summer at the regional scale, value with *** is significant at the 0.01 level; value with ** is significant at the 0.05 level; value with * is significant at the 0.1 level.

| **Region** | **EC** | **MUPR** | **MUYR** | **MYR** | **NC** | **NE** | **QTP** | **SC** | **UYR** | **XJ** |
|---|---|---|---|---|---|---|---|---|---|---|
| **Season** | Summer | Summer | Summer | Summer | Summer | Summer | Summer | Summer | Summer | Summer |
| **P_I_PM** | 0.06** | 0.10*** | 0.11*** | 0.11*** | 0.09*** | 0.09*** | 0.07 | 0.07** | 0.05 | 0.38*** |
| **P_P_PM** | 0.02 | 0.17*** | 0.06*** | 0.09*** | 0.09*** | 0.06** | 0.21*** | 0.19*** | 0.15*** | 0.23*** |
| **P_R_PM** | 0.06* | 0.12*** | 0.15*** | 0.03 | 0.26*** | 0.05* | 0.02 | 0.18*** | 0.01 | 0.52*** |
| **P_T_PM** | 0.12*** | 0.09*** | 0.18*** | 0.14*** | 0.11*** | 0.14*** | 0.04 | 0.02 | 0.07 | 0.54*** |
| **I_NO** | 0.18*** | 0.20*** | 0.17*** | 0.09 | 0.08** | 0.07** | 0.14** | 0.12*** | 0.08 | 0.09** |
| **P_NO** | 0.12*** | 0.16** | 0.19*** | 0.10*** | 0.11*** | 0.18*** | 0.24** | 0.15*** | 0.24*** | 0.07 |
| **R_NO** | 0.07 | 0.22*** | 0.16*** | 0.04 | 0.19*** | 0.13*** | 0.29*** | 0.16*** | 0.06 | 0.24*** |
| **T_NO** | 0.11*** | 0.17*** | 0.22*** | 0.21*** | 0.29*** | 0.12*** | 0.29*** | 0.12*** | 0.12* | 0.24*** |
| **I_VO** | 0.13*** | 0.19*** | 0.14*** | 0.07 | 0.06* | 0.11*** | 0.13** | 0.11*** | 0.08 | 0.09** |
| **P_VO** | 0.07** | 0.16*** | 0.10*** | 0.11*** | 0.15*** | 0.12*** | 0.03 | 0.08*** | 0.28*** | 0.06 |
| **R_VO** | 0.17*** | 0.17*** | 0.18*** | 0.12 | 0.29*** | 0.12** | 0.07 | 0.10 | 0.02 | 0.32*** |
| **T_VO** | 0.16*** | 0.22*** | 0.23*** | 0.17*** | 0.17*** | 0.14*** | 0.10* | 0.08 | 0.12* | 0.35*** |
| **A_NH** | 0.12*** | 0.20*** | 0.16*** | 0.19*** | 0.14*** | 0.20*** | 0.22*** | 0.16*** | 0.06 | 0.21*** |
| **I_NH** | 0.14*** | 0.15*** | 0.17*** | 0.09*** | 0.10*** | 0.09 | 0.35*** | 0.10 | 0.07 | 0.09** |
| **R_NH** | 0.14*** | 0.30*** | 0.17*** | 0.10** | 0.23*** | 0.14*** | 0.39*** | 0.30*** | 0.03 | 0.36*** |
| **T_NH** | 0.18*** | 0.16*** | 0.17*** | 0.22*** | 0.17*** | 0.12*** | 0.08 | 0.09* | 0.14** | 0.35*** |
| **I_SO** | 0.14*** | 0.12*** | 0.11*** | 0.10*** | 0.03 | 0.13*** | 0.31*** | 0.16*** | 0.10* | 0.09** |
| **P_SO** | 0.05 | 0.17* | 0.03 | 0.13*** | 0.12*** | 0.19*** | 0.24** | 0.20*** | 0.23*** | 0.06 |
| **R_SO** | 0.10* | 0.06** | 0.15*** | 0.08* | 0.15*** | 0.14*** | 0.30*** | 0.24*** | 0.24*** | 0.31*** |
| **T_SO** | 0.17*** | 0.17*** | 0.19*** | 0.21*** | 0.15*** | 0.13*** | 0.24*** | 0.05 | 0.14** | 0.41*** |

**Table S5.** Effect of various anthropogenic factors with different emission sectors on $PM_{2.5}$ concentrations in autumn at the regional scale, value with *** is significant at the 0.01 level; value with ** is significant at the 0.05 level; value with * is significant at the 0.1 level.

| Region | EC | MUPR | MUYR | MYR | NC | NE | QTP | SC | UYR | XJ |
|---|---|---|---|---|---|---|---|---|---|---|
| Season | Autumn | Autumn | Autumn | Autumn | Autumn | Autumn | Autumn | Autumn | Autumn | Autumn |
| P_I_PM | 0.02 | 0.17*** | 0.11*** | 0.08*** | 0.09*** | 0.06*** | 0.09 | 0.05 | 0.11*** | 0.24*** |
| P_P_PM | 0.00 | 0.28*** | 0.07*** | 0.07*** | 0.07*** | 0.05*** | 0.14** | 0.21*** | 0.03 | 0.14*** |
| P_R_PM | 0.02 | 0.07*** | 0.13*** | 0.11*** | 0.27*** | 0.18*** | 0.17 | 0.07 | 0.11*** | 0.47*** |
| P_T_PM | 0.05* | 0.10*** | 0.17*** | 0.10*** | 0.13*** | 0.05*** | 0.05 | 0.04 | 0.13*** | 0.41*** |
| I_NO | 0.14*** | 0.18*** | 0.17*** | 0.13*** | 0.09*** | 0.12*** | 0.10 | 0.12** | 0.20*** | 0.24*** |
| P_NO | 0.09** | 0.24*** | 0.16*** | 0.08** | 0.10*** | 0.10*** | 0.15* | 0.22*** | 0.14*** | 0.02 |
| R_NO | 0.06 | 0.14** | 0.19*** | 0.17*** | 0.22*** | 0.16*** | 0.20** | 0.10** | 0.17*** | 0.29*** |
| T_NO | 0.08*** | 0.23*** | 0.23*** | 0.13*** | 0.24*** | 0.08*** | 0.18*** | 0.11*** | 0.21*** | 0.31*** |
| I_VO | 0.16*** | 0.24*** | 0.15*** | 0.07* | 0.10*** | 0.06* | 0.08 | 0.18*** | 0.15*** | 0.22*** |
| P_VO | 0.05 | 0.29*** | 0.11*** | 0.10*** | 0.10*** | 0.05* | 0.05 | 0.19*** | 0.13*** | 0.05 |
| R_VO | 0.12*** | 0.14*** | 0.21*** | 0.30*** | 0.24*** | 0.33*** | 0.20 | 0.15*** | 0.32*** | 0.38*** |
| T_VO | 0.15*** | 0.16*** | 0.17*** | 0.11*** | 0.15*** | 0.08*** | 0.07 | 0.10** | 0.17*** | 0.35*** |
| A_NH | 0.09** | 0.13*** | 0.16*** | 0.09*** | 0.09*** | 0.05 | 0.15*** | 0.13** | 0.04 | 0.13*** |
| I_NH | 0.09** | 0.10*** | 0.12*** | 0.08*** | 0.06* | 0.04 | 0.16* | 0.44*** | 0.15*** | 0.22*** |
| R_NH | 0.12*** | 0.22*** | 0.17*** | 0.17*** | 0.30*** | 0.22*** | 0.22** | 0.18*** | 0.26*** | 0.34*** |
| T_NH | 0.15*** | 0.22*** | 0.16*** | 0.15*** | 0.15*** | 0.07** | 0.08 | 0.18*** | 0.17*** | 0.33*** |
| I_SO | 0.06 | 0.20*** | 0.11*** | 0.11*** | 0.06* | 0.09*** | 0.16* | 0.15*** | 0.20*** | 0.12*** |
| P_SO | 0.03 | 0.31*** | 0.05* | 0.10*** | 0.11*** | 0.10*** | 0.15 | 0.28*** | 0.06 | 0.02 |
| R_SO | 0.05 | 0.17*** | 0.20*** | 0.10*** | 0.23*** | 0.21*** | 0.19* | 0.25*** | 0.14*** | 0.39*** |
| T_SO | 0.15*** | 0.24*** | 0.21*** | 0.14*** | 0.15*** | 0.06** | 0.12*** | 0.09** | 0.18*** | 0.39*** |

**Table S6.** Effect of various anthropogenic factors with different emission sectors on $PM_{2.5}$ concentration in winter at the regional scale, value with *** is significant at the 0.01 level; value with ** is significant at the 0.05 level; value with * is significant at the 0.1 level.

| **Region** | **EC** | **MUPR** | **MUYR** | **MYR** | **NC** | **NE** | **QTP** | **SC** | **UYR** | **XJ** |
|---|---|---|---|---|---|---|---|---|---|---|
| **Season** | Winter | Winter | Winter | Winter | Winter | Winter | Winter | Winter | Winter | Winter |
| **P_I_PM** | 0.02 | 0.12*** | 0.15*** | 0.15*** | 0.10*** | 0.19*** | 0.24*** | 0.06* | 0.15*** | 0.36*** |
| **P_P_PM** | 0.03 | 0.24*** | 0.18*** | 0.11*** | 0.04** | 0.05** | 0.22*** | 0.15*** | 0.06** | 0.26*** |
| **P_R_PM** | 0.07** | 0.03 | 0.28*** | 0.07*** | 0.29*** | 0.06*** | 0.29** | 0.03 | 0.09*** | 0.13** |
| **P_T_PM** | 0.09*** | 0.12*** | 0.18*** | 0.21*** | 0.15*** | 0.19*** | 0.21*** | 0.03 | 0.15*** | 0.33*** |
| **I_NO** | 0.14*** | 0.10 | 0.16*** | 0.18*** | 0.10*** | 0.18*** | 0.28** | 0.04 | 0.21*** | 0.19*** |
| **P_NO** | 0.08 | 0.15** | 0.23*** | 0.11*** | 0.07*** | 0.12*** | 0.20** | 0.14*** | 0.08 | 0.15** |
| **R_NO** | 0.15*** | 0.04 | 0.30*** | 0.10*** | 0.19*** | 0.03 | 0.36*** | 0.06 | 0.10* | 0.43*** |
| **T_NO** | 0.13*** | 0.22*** | 0.27*** | 0.23*** | 0.25*** | 0.20*** | 0.16*** | 0.07 | 0.18*** | 0.40*** |
| **I_VO** | 0.16*** | 0.27*** | 0.15*** | 0.07* | 0.12*** | 0.22*** | 0.23** | 0.07 | 0.14** | 0.24*** |
| **P_VO** | 0.02 | 0.22*** | 0.14*** | 0.12*** | 0.07*** | 0.11*** | 0.20** | 0.11*** | 0.10*** | 0.23*** |
| **R_VO** | 0.11** | 0.05 | 0.32*** | 0.16*** | 0.30*** | 0.08*** | 0.34*** | 0.07 | 0.12*** | 0.43*** |
| **T_VO** | 0.12*** | 0.22*** | 0.17*** | 0.25*** | 0.19*** | 0.24*** | 0.24*** | 0.08 | 0.15*** | 0.41*** |
| **A_NH** | 0.18** | 0.12*** | 0.27*** | 0.24*** | 0.18*** | 0.12* | 0.26*** | 0.09 | 0.04 | 0.28*** |
| **I_NH** | 0.16*** | 0.12*** | 0.19*** | 0.14*** | 0.14*** | 0.17*** | 0.29** | 0.25*** | 0.20*** | 0.26*** |
| **R_NH** | 0.17*** | 0.13*** | 0.23*** | 0.16*** | 0.28*** | 0.19*** | 0.36*** | 0.11*** | 0.32*** | 0.30*** |
| **T_NH** | 0.24*** | 0.22*** | 0.18*** | 0.25*** | 0.20*** | 0.20*** | 0.26*** | 0.13** | 0.18*** | 0.49*** |
| **I_SO** | 0.02 | 0.13*** | 0.18*** | 0.17*** | 0.08*** | 0.26*** | 0.33** | 0.04 | 0.25*** | 0.19*** |
| **P_SO** | 0.05 | 0.27*** | 0.09*** | 0.17*** | 0.09** | 0.13*** | 0.21** | 0.19*** | 0.09*** | 0.18** |
| **R_SO** | 0.10* | 0.20*** | 0.27*** | 0.01 | 0.15*** | 0.02 | 0.37*** | 0.16*** | 0.12*** | 0.26*** |
| **T_SO** | 0.24*** | 0.24*** | 0.26*** | 0.24*** | 0.20*** | 0.18*** | 0.27*** | 0.06 | 0.16*** | 0.41*** |

**References:**

Li, X., Song, H., Zhai, S., Lu, S., Kong, Y., Xia, H., Zhao, H., 2019. Particulate matter pollution in Chinese cities: Areal-temporal variations and their relationships with meteorological conditions (2015–2017). Environmental Pollution. 246, 11-18.
